# Exploring gender and ethnic disparities in sarcoidosis: insights from the British Thoracic Society UK Interstitial Lung Disease Registry

**DOI:** 10.1136/bmjresp-2025-003449

**Published:** 2025-11-18

**Authors:** Karol Kamil Bączek, Sia Leng Cheng, Gina Amanda, Andrew Achaiah, Lucile Sesé, Nazia Chaudhuri, British Thoracic Society Interstitial Lung Disease Registry Working Group, Nazia Chaudhuri

**Affiliations:** 1 Medical University of Lodz, Lodz, Poland; 2 Department of Medicine, Faculty of Medicine, University Malaya Medical Centre, Kuala Lumpur, Malaysia; 3 Jakarta Islamic Hospital Cempaka Putih, Jakarta, Indonesia; 4 Gloucestershire Hospitals NHS Foundation Trust, Gloucestershire, UK; 5 Respiratory Department, Avicenne Hospital, AP-HP, Bobigny, France; 6 Physiology Department, Avicenne Hospital, AP-HP, Bobigny, France; 7 School of Medicine, University of Ulster Faculty of Life and Health Sciences, Londonderry, UK

**Keywords:** Sarcoidosis, Clinical Epidemiology

## Abstract

**Introduction:**

Sex and ethnicity influence sarcoidosis internationally, but UK data are limited. We analysed the British Thoracic Society Interstitial Lung Disease Registry to assess whether gender or ethnic differences affect presentation and management of pulmonary sarcoidosis in the UK.

**Methods:**

A retrospective study included adults with confirmed pulmonary sarcoidosis recorded between January 2013 and December 2024. Demographics, symptoms, comorbidities, investigations, radiology, treatment and Index of Multiple Deprivation were extracted. Group comparisons used χ², t-tests or Mann-Whitney U tests; multivariable logistic regression identified factors associated with immunosuppressive initiation.

**Results:**

Among 1071 patients, 55.5% were male; median age 54 years (SD 13). Ethnicity was documented in 918 (85.7%): 55.4% white, 14.2% non-white (black, South Asian, mixed).

Gender: Women presented older than men (56 vs 52 years; p=0.002) and reported more fatigue, higher erythrocyte sedimentation rate and prior tuberculosis. Men had more lymphopenia, elevated ACE and arrhythmia. Lung function and CT patterns were similar, but methotrexate use was higher in men (4.9% vs 2.3%; p=0.017).

Non-white patients presented younger (52 vs 54 years; p<0.001) with greater symptom burden (breathlessness 46% vs 33%; cough 44% vs 27%) and more comorbidities (hypertension, diabetes, tuberculosis). Physiology was comparable, but CT nodularity (54% vs 36%) and abnormal liver tests (16% vs 9%) were more frequent, and mycophenolate was prescribed more often (3.7% vs 0.3%; p=0.036).

In multivariable analysis, male sex (OR 2.34), non-white ethnicity (OR 3.20), breathlessness (OR 2.05) and lower forced vital capacity (OR 0.97 per % predicted) were independently associated with immunosuppressive treatment (all p≤0.031).

**Conclusions:**

In this UK cohort, treatment decisions were more influenced by sex and ethnicity than by lung function or imaging. Male and non-white patients received immunosuppression more frequently, suggesting possible biological, socioeconomic or practice-related differences.

**WHAT IS ALREADY KNOWN ON THIS TOPIC**

- Sex and ethnicity-related disparities in sarcoidosis presentation and management have been described in North American and Scandinavian cohorts, but UK-specific evidence has been limited.

**WHAT THIS STUDY ADDS**

- In the largest UK sarcoidosis dataset to date, male sex and non-white ethnicity were associated with higher odds of receiving immunosuppressive therapy—despite comparable lung function and imaging—highlighting distinct demographic phenotypes that influence treatment decisions.

**HOW THIS STUDY MIGHT AFFECT RESEARCH, PRACTICE OR POLICY**

- Recognising these inequities should prompt prospective, equity-focused research and guide UK clinicians and policy-makers to incorporate demographic-aware decision tools, fostering more personalised and consistent sarcoidosis care.

## Introduction

 Sarcoidosis is a multisystem granulomatous inflammatory disorder affecting many organs, most commonly the lungs, skin, eyes and lymphatic system.^1^ The aetiology of sarcoidosis remains unclear, where genetic variants (eg, HLA-DRB1*1101; ANXA11)^2 3^ and environmental exposure (eg, silica, metal dusts)^4^ are implicated in disease progress.^5^,^7^ The annual incidence of sarcoidosis varies across continents, ranging from 1 to 15 per 100 000 people. The lowest incidences are in Eastern Asian countries (1–3 per 100 000 people),^8^ whereas the highest rates are found in Northern Europe, mainly in Scandinavian countries (10–20 per 100 000 people).^9^,^11^

Several factors, including age, gender, ethnicity and geographical region, contribute to the occurrence, clinical manifestation and prognosis of patients with sarcoidosis.^5 12^ Studies in Japan and the USA have found sarcoidosis to be more prevalent among females (1.4–6.3 females vs 1.2–5.9 males per 100 000 people).^713^,^15^ Conversely, studies in Sweden and Denmark have demonstrated that males were predominant.^16^,^18^ Females have late-onset disease and higher mortality rates compared with males.^7 19 20^ Gender differences are also seen with respect to organ involvement.^7 16 21^ Pulmonary and cardiac involvement is more frequent among males (eg, 36% vs 46% in males with pulmonary involvement), while females are more likely to present with peripheral lymph nodes, salivary glands, skin, eyes and liver involvement (eg, 13% vs 8% in females with skin involvement).^7 16^

The aetiology of gender differences may be related to sex hormones, genetic, epigenetic and environmental exposures.^19^ Sex hormones (oestrogen) may play a role since sarcoidosis is more commonly found in post-menopausal women.^7 22 23^ Additionally, pregnancy can improve the disease due to immunological changes during that period, such as a switch from Th1-dependent into Th2-dependent response.^24^ In contrast, hormonal replacement therapy has been attributed to worsening of disease.^25^ A study in Sweden showed a genetic association between gender groups in sarcoidosis. Xiong *et al* identified gender-dependent genetic variation influenced the phenotype of sarcoidosis patients with Löfgren syndrome such as MHC-C6orf10 in females.^19^ Sarcoidosis is also influenced by occupational and environmental exposure, so gender-biased activities and occupation may increase the rate of sarcoidosis (eg, male predominance in firefighters and police officers exposed to World Trade Center catastrophe).^7^

Ethnic variations also exist in the epidemiology of sarcoidosis. Studies in several countries showed that the incidence of sarcoidosis was higher among the Black ethnicity. In the USA, the incidence of sarcoidosis was 17.8 in African Americans, 8.1 in whites, 4.3 in Hispanics and 3.2 in Asians per 100 000 of the population. Ethnicity disparities may result from genetics (eg, ANXA11 mutation is more prevalent in African American sarcoidosis patients)^3^ and socioeconomic status. Low income and public insurance in black Americans result in more severe pulmonary and extrapulmonary disease (up to 70% of African American patients may present with extrapulmonary involvement vs 50% of white American sarcoidosis patients).^26^,^28^

From these studies, it is evident that gender and ethnicity may influence the presentation and outcomes of sarcoidosis, but that this can vary according to geographical location. We thus sought to ascertain whether there are gender and ethnicity differences in the presentation and outcomes of sarcoidosis in the UK.

## Methods

### Study design and data source

This was a retrospective observational study using data from the British Thoracic Society (BTS) UK Interstitial Lung Disease Registry. This registry was established in 2013 with 91 hospital sites across the UK contributing patient data. Data entry was collected through a custom-designed web-based platform at https://registry.brit-thoracic.org.uk/. Written and informed consents were obtained by all patients. Data access was granted through data-sharing agreements. Grants of the registry were provided by the Healthcare Quality Improvement Partnership, Boehringer Ingelheim and InterMune between 2011 and 2014.

### Study population

The study included patients with a confirmed diagnosis of pulmonary sarcoidosis from the national registry between January 2013 and 2024. ILD diagnoses other than sarcoidosis were excluded. Ethnicity was recorded using routine categories. Because several detailed non-white subgroups had small cell counts (eg, East Asian/Chinese and Mixed), our primary analyses compared non-white versus white to preserve power and control type-I error. To enhance transparency, stratified descriptives for black African, black Caribbean, South Asian (Indian/Pakistani/Bangladeshi), East Asian/Chinese and mixed are provided in online supplemental tables.

### Baseline assessment

Baseline demographic variables were collected and are presented in table 1. Also, lung biopsy results and treatment history were recorded. The Index of Multiple Deprivation (IMD) 2019 was provided. The IMD was divided into quintiles and classification is based on seven deprivation domains: income, employment, education, health, crime, housing and services, and living environment, reflecting the socioeconomic status of the population.^19 29^

**Table 1:** Baseline variables collected in the registry

Demographics	Symptoms	Duration of symptoms	Comorbidities	Lung function tests	HRCT findings	Laboratory findings
Sex	Subcutaneous nodules	≥12 months	Diabetes	FVC	Traction bronchiectasis	Raised IgG
Age	Fatigue	<12 months	Hypertension	DLCO	Reticulation	Raised ESR
Ethnicity	Musculoskeletal pain		Ischaemic heart disease		Cysts	Raised CRP
Smoking status	Fever		Tuberculosis		Honeycombing	Raised Calcium
IMD	Breathlessness		Depression		Nodules	Abnormal liver function
	Cough		Arrhythmia		Ground glass density	Abnormal renal function
	Erythema nodosum		Gastro-oesophageal reflux disease		Consolidation	Lymphopenia
	Cardiac symptoms					Thrombocytopenia
	Eye symptoms					

CRP, C reactive protein; DLCO, diffusing capacity of the lung for carbon monoxide; ESR, erythrocyte sedimentation rate; FVC, forced vital capacity; HRCT, high resolution CT; IgG, Immunoglobulin G; IMD, Index of Multiple Deprivation.

### Outcome measures

The differences between gender and ethnicity in baseline characteristics, duration of chest symptoms, laboratory findings, lung function test, radiological patterns, lung biopsy and treatment outcome were compared. Overall survival was defined in years from date of diagnosis to date of death from any cause. The date of presentation to the chest clinic was taken as the date of diagnosis.

### Data analysis

Statistical analyses were performed using SPSS V.29.0.2.0 (20). Continuous variables were reported as mean±SD or median with IQR as appropriate, while categorical variables were presented as numbers and percentages. Group comparisons were conducted using χ² tests for categorical variables, independent t-tests for normally distributed continuous variables and Mann-Whitney U tests for skewed data. A p<0.05 was considered statistically significant. Multivariate logistic regression analyses were performed. Multivariable logistic regression was used to identify independent predictors of treatment initiation. Covariates were prespecified and included sex, age, ethnicity, forced vital capacity (FVC) % predicted, diffusing capacity of the lung for carbon monoxide (DLCO %) predicted, smoking status, laboratory findings (raised erythrocyte sedimentation rate (ESR), raised ACE) and symptoms (breathlessness, musculoskeletal pain, erythema nodosum). We report adjusted ORs with 95% CIs. Receiver operating characteristic (ROC) curves were generated to evaluate discrimination (area under the curve, AUC). A p<0.05 was considered statistically significant for all tests.

### Missing data

We assessed completeness for all variables. Descriptive denominators reflect available data. Multivariable models used a complete-case approach; sensitivity analyses with missing-indicator categories for key predictors (smoking, lung function) were directionally consistent. Because missingness in smoking status differed by sex, we note that differential documentation may bias sex comparisons and should be considered when interpreting findings. Given the extent and differential patterns of missingness (eg, smoking by sex), external generalisability may be limited; however, complete-case estimates were directionally consistent with sensitivity analyses using missing-indicator categories.

### Patient and public involvement

Patients and/or the public were not involved in the design, conduct, reporting or dissemination plans of this research.

## Results

### Baseline patient characteristics

A total of 1071 patients with sarcoidosis were included, of whom 595 (55.5%) were male and 398 (37.2%) were female (M:F ratio 1.5:1; online supplemental table 1). In 78 (7.3%) records, gender was not recorded. Among those with recorded data on ethnicity (918, 85.7%), 594 (55.5%) were white, while the remaining comprised other ethnic backgrounds (black African/Caribbean 62 (5.8%), South Asian 69 (6.4%), mixed ethnicity 20 (1.9%) and East Asian/Chinese 1 (0.1%)). In 172 (16%), ethnicity was not stated. The mean (±SD) age at presentation was 54 (±13) years. Current or former smokers accounted for 19.2% the cohort, whereas 28.7% had never smoked, and in 46.4% smoking status was not recorded.

### Symptoms

A total of 924 symptoms were reported in the cohort. 85 (8%) patients were explicitly coded as asymptomatic at presentation, whereas 80 (7.5%) records had the symptoms field marked as ‘not recorded’. 177 (16.5%) patients had one symptom, 150 (14.0%) had two symptoms, 80 (7.5%) had three symptoms and 72 (6.7%) had over four symptoms. The most frequently reported symptoms were breathlessness (272 (25.4%)), cough (231 (21.6%)) and fatigue (130 (12.1%)).

### Comorbidities

At least one comorbidity was present in 15.5% of cases and 26.6% had no comorbidities; the most prevalent comorbidities were hypertension (7.9%), diabetes (6.6%) and ischaemic heart disease (1.9%). In 620 patients (57.9%), comorbidities were not documented.

### Laboratory findings/pulmonary function tests

Common laboratory findings included lymphopenia (174 (16.2%)), abnormal liver function (80 (7.5%)) and elevated angiotensin-converting enzyme levels (75 (7.0%)); 631 (58.9%) had no recorded abnormalities and 9.1% of cases were not documented. Lung function FVC was available in 47.2% and DLCO in 36.7% of the cohort. The median FVC was 3.60 L (95% CI 0.99 to 7.48), corresponding to a median of 98.44% predicted. The median DLCO was 7.15 mmol/min/kPa (95% CI 1.28 to 14.30), with a median of 79.72% predicted.

### Radiology

Radiologically, nodules were observed in 295 (27.5%) cases, ground-glass opacities in 51 (4.8%) and traction bronchiectasis in 38 (3.5%). 684 (63.9%) had no abnormalities. A biopsy was performed in 42 (4.2%) patients, 91.8% of cases were not documented.

### Factors associated with immunosuppressive treatment

First-line therapies (prednisolone at both high (≥10 mg/daily) and low doses (<10 mg/daily) or methylprednisolone) were administered to 228 (21.3%) patients, while second-line treatments (methotrexate (MTX), azathioprine, hydroxychloroquine, mycophenolate mofetil (MMF)) were recorded in 60 (5.6%) cases. 345 (32.2%) patients had no immunosuppressive treatments and in 470 (43.9%) data on treatment was not documented. There was no statistically significant difference in patients’ demographics between treated and untreated patients. Patients with musculoskeletal pain (56% vs 42%; p=0.008), erythema nodosum (62% vs 42%; p=0.003) and breathlessness (55% vs 35%; p<0.001) were significantly more likely to be treated with immunosuppression than no treatment. In contrast, patients with elevated ACE levels and raised ESR were less likely to receive immunosuppressive treatment (raised ACE: 32% vs 45%; p=0.025) (raised ESR: 22% vs 45%; p=0.008).

Furthermore, those treated with immunosuppression had lower FVC percent predicted (92% vs 100%; p<0.001), DLCO percent predicted (77% vs 81%; p=0.006) and greater extent of traction bronchiectasis (68% vs 47%; p=0.008). All detailed information was described in online supplemental table 1.

### Gender differences in the UK sarcoidosis cohort

Our patient cohort consisted of 55.5% men and 37.2% women (table 2). The median age at diagnosis was significantly higher among women compared with men (56 vs 52 years; p=0.002). Women were more likely to present with a shorter duration of symptoms (<6 months: 2.2 vs 1.0%; p=0.040), although there was poor documentation of symptom duration with almost 92% of patients not having symptom duration documented. Women also reported fatigue more frequently than men (15.8% vs 11.3%; p=0.05). Most patients were current or former smokers, with a markedly higher proportion among men than women (22.4% vs 18.3%; p=0.013). Conversely, the percentage of never-smokers was greater among women than men (41.9% vs 33.8%; p=0.013). Although women were more often never-smokers, men had a higher proportion of missing smoking data (43.8% vs 39.7%; p=NS). This likely reflects documentation practices and could bias apparent sex differences in smoking status.

**Table 2:** Comparison of significant characteristics of sarcoidosis patients by sex

	Sex		P value
	Male n=594 (55.5%)	Female n=398 (37.2%)	NS
Sex—missing data	N=78 (7.3%)		
Age, years (median (IQR))	52 (42–61)	56 (46–63)	**0.002**
Smoking status			
Current/ex-smoker (%)	133 (22.4%)	73 (18.3%)	**0.013**
Never (%)	201 (33.8%)	167 (41.9%)	**0.013**
Symptoms			
Fatigue (%)	67 (11.3%)	63 (15.8%)	**0.05**
Duration of symptoms prior to chest clinic			
<6 months (%)	6 (1.0%)	9 (2.2%)	**0.040**
Comorbidities			
Arrhythmia (%)	6 (1.0%)	0 (0.0%)	**0.042**
Tuberculosis (%)	1 (0.2%)	7 (1.7%)	**0.009**
Lung function			
FVC % (median (IQR))	96.1 (91.8–97.0) N=241 (40.6%)	100.4 (95.1–102.1) N=151 (37.9%)	NS
DLCO % (median (IQR)	82.7 (78.2–83.0) N=241 (40.6%)	77.7 (74.3–80.7) N151 (37.9%)	NS
Blood test results			
Lymphopenia (%)	118 (19.9%)	56 (14.1%)	**0.002**
Raised ACE (%)	51 (8.6%)	24 (6.0%)	**0.05**
Raised ESR (%)	11 (1.8%)	21 (5.3%)	**0.003**
Treatment			
First-line glucocorticosteroids (%)	141 (23.7%)	98 (24.6%)	NS
Second-line immunosuppressants (%)	40 (6.7%)	20 (5.0%)	NS
Methotrexate (%)	29 (4.9%)	9 (2.3%)	**0.017**

Lung-function *n* counts represent participants with data available; those with missing values are excluded from denominators for those rows.

Significant p-values are presented in bold.

DLCO, diffusing capacity of the lung for carbon monoxide; ESR, erythrocyte sedimentation rate; FVC, forced vital capacity; NS, not significant.

Tuberculosis and arrhythmia were infrequently reported in the overall cohort at 0.7% and 0.6%, respectively. Tuberculosis as a comorbidity was more frequently reported in women than men (1.7% vs 0.2%; p=0.009), whereas arrhythmias were more common among men than women (1.0% vs 0.0%; p=0.042). No notable gender-based differences emerged in the prevalence of diabetes, ischaemic heart disease or hypertension.

Raised ESR was significantly more prevalent among women than men (5.3% vs 1.8%; p=0.003), whereas men had higher rates of elevated ACE levels (8.6% vs 6.0%; p=0.05) and lymphopenia than women (19.9% vs 14.1%; p=0.002). There was no difference in FVC, DLCO or radiological findings between genders.

In terms of treatment, men were more frequently treated with MTX (4.9% vs 2.3%; p=0.017). All detailed information was included in online supplemental tables 2 and 4.

Multivariate logistic regression—including sex, age, race, FVC, DLCO, smoking status, laboratory findings (raised ESR and ACE), and symptoms (breathlessness, musculoskeletal pain, erythema nodosum)—confirmed that lower FVC (OR=0.97, 95 % CI 0.95 to 0.98, p<0.001—lower FVC was associated with higher odds of immunosuppressive initiation), male sex (OR=2.34, 95 % CI 1.24 to 4.39, p=0.008), non-white ethnicity (OR=3.20, 95 % CI 1.11 to 9.22, p=0.031) and breathlessness (OR=2.05, 95 % CI 1.07 to 3.94, p=0.031) were independent predictors of initiating immunosuppressive treatment (table 3). ROC analysis further highlighted the discriminative power of these variables, with breathlessness showing the highest (AUC=0.602). By contrast, other factors—such as sex, ethnicity and FVC—exhibited weaker discriminatory performance.

**Table 3:** Multivariate logistic regression results of risk factors for initiation of immunosuppressive treatment

Variable	OR	95% CI	P value
FVC	0.97	0.95 to 0.98	**<0.001**
Male	2.34	1.24 to 4.39	**0.008**
Non-white	3.20	1.11 to 9.22	**0.031**
Breathlessness	2.05	1.07 to 3.94	**0.031**

For FVC, the OR is expressed per 1%-predicted increase (ie, lower FVC corresponds to higher odds of treatment initiation).

Significant p-values are presented in bold.

FVC, forced vital capacity.

### Ethnicity differences in the UK sarcoidosis cohort

Our patient cohort consisted predominantly of white individuals (55.4%), with non-white (black African, black Caribbean, Indian, Pakistani, Bangladeshi, South Asian and mixed) individuals accounting for 14.2% of the total sample (table 4). While our primary comparison is non-white versus white due to small subgroup sizes of non-whites, additional table documents heterogeneity across black African, black Caribbean, South Asian, East Asian/Chinese and mixed groups (online supplemental table 3). The median age at diagnosis was significantly higher among white individuals compared with non-whites (54 vs 52 years; p<0.001). Breathlessness, cough, fatigue, erythema nodosum and fever were all reported more frequently by non-white than by white individuals (46.0% vs 33.2%, p=0.003; 44.1% vs 27.4%, p<0.001; 27.6% vs 14.1%, p<0.001; 15.8% vs 6.6%, p<0.001; 5.9% vs 1.8%, p=0.004, respectively), and a higher percentage of non-white individuals reported no symptoms (17.1% vs 9.4%; p=0.006). Hypertension, diabetes and tuberculosis were all significantly more frequent among non-white than white individuals (18.3% vs 9.4%, p=0.004; 18.3% vs 7.1%, p<0.001 and 4.2% vs 0.3%, p<0.001, respectively), and a higher percentage of non-white individuals had no comorbidities (58.4% vs 33.0%; p<0.001).

**Table 4:** Comparison of significant characteristics of sarcoidosis patients by ethnicity

	Ethnicity		P value
	White N=594 (55.5%)	Non-white N=152 (14.2%)	<0.001
Ethnicity—missing data	N=153		
Ethnicity—not stated	N=172		
Age, years (median, (IQR))	54 (44–62)	52 (42–61)	**<0.001**
Smoking status			
Current/ex-smoker (%)	143 (24.1%)	56 (10.2%)	**<0.001**
Never (%)	213 (35.8%)	85 (15.7%)	**<0.001**
Not known (%)	26 (11.3%)	34 (12.0%)	**<0.001**
Missing data (%)	55 (9.2%)	37 (24.3%)	**<0.001**
Symptoms			
Breathlessness (%)	197 (33.2%)	70 (46.0%)	**0.003**
Cough (%)	163 (27.4%)	67 (44.1%)	**<0.001**
Fatigue (%)	84 (14.1%)	42 (27.6%)	**<0.001**
Erythema nodosum (%)	39 (6.6%)	24 (15.8%)	**<0.001**
Fever (%)	11 (1.8%)	9 (5.9%)	**0.004**
None (%)	56 (9.4%)	26 (17.1%)	**0.006**
Duration of symptoms prior to chest clinic			
<6 months (%)	9 (1.5%)	6 (3.9%)	**0.05**
Missing data (%)	577 (97.1%)	139 (91.4%)	**<0.001**
Comorbidities			
Hypertension (%)	56 (9.4%)	26 (18.3%)	**0.004**
Diabetes (%)	42 (7.1%)	26 (18.3%)	**<0.001**
Tuberculosis (%)	2 (0.3%)	6 (4.2%)	**<0.001**
None (%)	196 (33.0%)	83 (58.4%)	**<0.001**
Missing data (%)	264 (44.4%)	31 (21.8)	**<0.001**
Blood test results			
Abnormal liver function (%)	55 (9.2%)	25 (16.4%)	**0.009**
No abnormalities (%)	389 (65.5%)	68 (17.1%)	**0.004**
Radiological findings			
Nodules (%)	213 (35.8%)	82 (53.9%)	**0.013**
No abnormalities (%)	262 (44.1%)	27 (17.8%)	**<0.001**
Lung function			
FVC % (median, (IQR))	98.4 (94.6 to 99.4) N=247 (41.6%)	98.4 (90.5 to 98.4) N=110 (72.4%)	NS
DLCO % (median, (IQR))	80.0 (77.7 to 82.3) N=247 (41.6%)	77.7 (74.1 to 81.1) N=110 (72.4%)	NS
Treatment			
First-line glucocorticosteroids (%)	157 (26.4%)	77 (23.8%)	NS
Second-line immunosuppressants (%)	37 (6.2%)	22 (6.8%)	NS
Mycophenolate mofetil (%)	2 (0.3%)	5 (3.7%)	**0.036**
No treatment (%)	233 (39.2%)	45 (29.6%)	NS
Index of Multiple Deprivation Quintiles (IMQ)			
IMQ1 most deprived (%)	112 (18.8%)	71 (21.9%)	**0.022**

Primary ethnicity contrast is non-white versus white owing to small subgroup counts; stratified subgroup descriptives are provided in online supplemental table S3. Lung-function *n* counts represent participants with data available; those with missing values are excluded from denominators for those rows.

Significant p-values are presented in bold.

DLCO, diffusing capacity of the lung for carbon monoxide; FVC, forced vital capacity.

Non-white individuals more frequently presented with abnormal liver function (16.4% vs 9.2%; p=0.009). They also had a higher proportion of ‘no abnormalities’ (74.7% vs 65.5%; p=0.004). In terms of radiological findings, nodules were more prevalent in the non-white group (53.9% vs 35.8%; p=0.013), and normal radiological appearances in CT were more prevalent in the white group (44.1% vs 17.8%; p<0.001). In terms of lung function, results were similar.

First-line glucocorticoid therapy was given at similar rates in both groups (26.4% vs 23.8%; p=0.360), while MMF was used more frequently among non-white patients (3.7% vs 0.3%; p=0.036). Finally, in relation to socioeconomic factors (IMD Quintiles), non-white patients appeared more frequently in the most deprived quintile (21.9% vs 18.8%; p=0.022). All information about differences between white versus non-white was presented in online supplemental table 4.

## Discussion

Our analysis of 1071 patients enrolled in the BTS Sarcoidosis registry demonstrated an association between demographic factors—particularly sex and ethnicity, and differences in presentations and treatment decisions more than lung physiology or radiologic stage. Men represented 55.5 % of recorded cases and non -white patients 14.2%. The median age at diagnosis was 4 years older in women than men with distinct immunological presentations, with higher rates of raised ESR in women and conversely, men had higher rates of raised ACE and lymphopenia. Despite equivalent physiological and radiological findings between genders, women were less likely to be treated with MTX than males, with male gender being an independent predictor of the initiation of immunosuppression therapy.

Conversely, in non-white individuals, the median age at diagnosis was 2 years younger than white individuals. Breathlessness, cough, fatigue, erythema nodosum and fever were reported more frequently by non-white individuals. Non-white individuals had a higher frequency of abnormal liver abnormalities, nodules on CT imaging and were more likely to receive MMF. The reasons for these differences are complex and may raise the possibility of cultural preferences, clinician bias (conscious or not), or systemic barriers influencing presentations and management and highlight the need for individualised patient-centred approaches in the management and diagnosis of sarcoidosis.

Beyond documentation effects, our sex differences in smoking align with epidemiologic evidence that current smoking is inversely associated with sarcoidosis incidence compared with never smoking,^30 31^ which can make cohorts with a higher proportion of never-smokers (here, women) appear to carry more cases. In the UK, men are more likely to be current smokers than women,^32^ and they also consult primary care less often,^33^ reducing opportunities to record or update smoking status and plausibly contributing to the higher proportion of missing smoking data among men.

Although our findings reveal a male predominance (M:F≈1.5:1), this contrasts with the female preponderant pattern seen in the USA and Japan, where postmenopausal women dominate case series.^5^,^719 24^ Conversely, our sex distribution mirrors reports from Northern Europe and Scandinavia that describe higher or near-equal rates in men.^16 17 36^ Such regional differences may reflect heterogeneous genetic backgrounds (eg, HLADRB1*1101, ANXA11), hormonal influences (oestrogen or environmental exposures (silica, metal dusts).^23 7 10 11 19 25 26 34 37^,^39^

Notably, US studies frequently report that minoritised/non-white patients present later and with more advanced sarcoidosis compared with white patients, with greater extrapulmonary involvement and healthcare utilisation markers of severity.^2627 40^,^43^ These differences may reflect intersecting structural factors (insurance status, access, cumulative exposure burden) beyond biology. In our UK cohort—embedded in a tax-funded system—we observed younger age at presentation and higher symptom burden in non-white patients, but without systematically later physiological stage, underscoring the role of system-level context when comparing cohorts internationally.

Age at presentation diverged by sex: women were diagnosed at a median of 56 years vs 52 years in men, consistent with late-onset sarcoidosis after menopause.^7 19 25 26 28^ Immunological and endocrine shifts with ageing—and pregnancy- related fluctuations—may modulate disease expression.^7 24 25^

Ethnicity remained an important determinant. Non-white patients presented earlier and reported breathlessness, cough, fatigue, erythema nodosum and fever more frequently than white patients, echoing studies that document heavier sarcoid burdens in minority populations.^5 12 26 27 40 44 45^ Hypertension, diabetes and tuberculosis also clustered in non-white groups, underscoring the influence of social determinants of health.^26 27 39 40 46^ Despite this higher symptom burden—one of the principal indications for commencing sarcoidosis therapy—there were no statistically significant differences in treatment initiation or intensity between non-white and white patients.

Laboratory profiles retained the sex- linked patterns reported previously: elevated serum ACE and lymphopenia were commoner in men, whereas raised ESR predominated in women. Non-white individuals more often had abnormal liver function tests. These findings point to subtle immunophenotypic differences that warrant mechanistic study.^6 7 25 47^

These findings underscore the need for heightened awareness in UK practice of how gender and ethnicity modulate sarcoidosis presentation and treatment pathways, prompting clinicians to scrutinise decision-making for potential inequities and to tailor patient counselling accordingly. Importantly, lung function (FVC, DLCO) and thoracic CT findings did not differ significantly between sexes or ethnicities, suggesting that parenchymal involvement may evolve similarly once sarcoidosis is established. Nonetheless, prior work describes heterogeneous lung-function phenotypes by race and sex.^48^ Yet treatment patterns diverged: women were less likely than men to receive MTX (2.3% vs 4.9%), and non-white patients were more likely to receive MMF (3.7% vs 0.3%). Multivariable modelling confirmed male sex, non-white ethnicity, lower FVC and breathlessness as independent predictors of immunosuppressive initiation. Whether these prescribing differences reflect clinician preference, patient choice, pharmacogenetic factors or unconscious bias merits prospective investigation—especially because they persist despite non-white patients reporting a greater symptom burden—so that future guidelines can promote truly equitable, personalised care.** **

Limitations include incomplete documentation for symptom duration, lung function, radiology and multidisciplinary input, which may temper generalisability. Moreover, the registry itself has evolved over time; some variables—especially certain comorbidities—were not explicitly collected in earlier iterations, leading to artificially low frequencies in the current dataset. Nevertheless, our findings highlight the continuing need for personalised pathways that account for demographic context. Future longitudinal studies incorporating genomics, epigenetics and patient- reported outcomes should clarify why comparable physiological disease can prompt divergent therapeutic strategies.^7 26^A more nuanced understanding of how demographic and biological factors intersect in sarcoidosis will refine clinical decision-making and improve outcomes.

## Supplementary material

10.1136/bmjresp-2025-003449 online supplemental file 1

10.1136/bmjresp-2025-003449 online supplemental file 2

10.1136/bmjresp-2025-003449 online supplemental file 3

## Data Availability

Data are available on reasonable request.
