# Supplementary material for: Exploring gender and ethnic disparities in sarcoidosis: insights from the British Thoracic Society UK Interstitial Lung Disease Registry
Source: BMJ Open Respir Res. 2025 Nov 18;12(1):e003449. doi: 10.1136/bmjresp-2025-003449 (PMC12636952; doi:10.1136/bmjresp-2025-003449)
Supplement: online supplemental file 1 [file bmjresp-12-1-s001.pdf]

Supplementary table 1. Baseline data for the whole sarcoidosis cohort

**Gender**	
Males (%)	595 (55.5%)
Females (%)	398 (37.2%)
Not documented (%)	78 (7.3%)
**Age, years (mean ±SD)**	54 ±13
**Ethnicity**	
White (%)	594 (55.5%)
Not stated (%)	172 (16.0%)
South Asian/Indian/Pakistani/Bangladeshi (%)	69 (6.4%)
Black African/Caribbean (%)	62 (5.8%)
Mixed (%)	20 (1.9%)
East Asian/Chinese (%)	1 (0.1%)
Missing data (%)	153 (14.3%)
**Smoking status**	
Current or former (%)	206 (19.2%)
Never (%)	307 (28.7%)
Not known (%)	61 (5.7%)
Missing data (%)	497 (46.4%)
**Symptoms**	
Symptoms present (%)	479 (44.7%)
Breathlessness (%)	272 (25.4%)
Cough (%)	231 (21.6%)
Fatigue (%)	130 (12.1%)
Musculoskeletal Pain (%)	93 (8.7%)
Eye symptoms (%)	71 (6.6%)
Erythema Nodosum (%)	63 (5.9%)
Cardiac symptoms (%)	24 (2.2%)
Fever (%)	20 (1.9%)
Subcutaneous Nodules (%)	20 (1.9%)
No symptoms (%)	80 (7.9%)
Missing data (%)	504 (47.1%)
**Duration of symptoms**	
≥12 months (%)	21 (2.0%)
<12 months (%)	25 (2.3%)
Not known (%)	36 (3.3%)
Missing data (%)	989 (92.3%)
**Comorbidities**	
Hypertension (%)	85 (7.9%)
Diabetes (%)	71 (6.6%)
Ischemic heart disease (%)	20 (1.9%)
GERD (%)	12 (1.1%)
TB (%)	8 (0.7%)
Malignancy (%)	7 (0.7%)
Arrythmia (%)	6 (0.6%)
Depression (%)	6 (0.6%)
No comorbidities (%)	285 (26.6%)
Missing data (%)	620 (57.9%)

Laboratory findings	
Lymphopenia (%)	174 (16.2%)
Abnormal liver function (%)	80 (7.5%)
Raised ACE (%)	75 (7.0%)
Raised CRP (%)	44 (4.1%)
Raised ESR (%)	32 (3.0%)
Raised Ca (%)	22 (2.1%)
Abnormal renal function (%)	12 (1.1%)
Raised IgG (%)	9 (0.8%)
Thrombocytopenia (%)	4 (0.4%)
Not recorded (%)	90 (8.4%)
No abnormalities (%)	631 (58.9%)
Radiological findings	
Nodules (%)	295 (27.5%)
Ground glass density (%)	51 (4.8%)
Traction bronchiectasis (%)	38 (3.5%)
Normal (%)	32 (3.0%)
Consolidation (%)	16 (1.5%)
Reticulation (%)	6 (0.6%)
Honeycombing (%)	5 (0.5%)
Cysts (%)	4 (0.4%)
No abnormalities (%)	684 (63.9%)
Pulmonary function tests results	
FVC Absolute - median [IQR, 95% CI]	3.60 L [IQR 1.63, 0.99–7.48] N=505 (47.2%)
FVC Predicted %	98.44%
DLCO Absolute - median [IQR, 95% CI]	7.15 mmol/min/kPa [IQR 3.18, 95% CI 1.28–14.30] N=393 (36.7%)
DLCO Predicted %	79.72%
Treatment	
First-line treatment (%)	228 (21.3%)
High dose Prednisone (%)	212 (19.8%)
Low dose Prednisone (%)	9 (0.8%)
IV Methyl prednisone (%)	7 (0.7%)
Second-line treatment (%)	60 (5.6%)
Methotrexate (%)	38 (3.5%)
Hydroxychloroquine (%)	10 (0.9%)
Mycophenolate mofetil (%)	7 (0.7%)
Azathioprine (%)	6 (0.6%)
No treatment (%)	345 (32.2%)
Missing data (%)	470 (43.9%)

Lung-function *n* counts represent participants with data available; those with missing values are excluded from denominators for those rows

Supplementary table 2. Comparison of characteristics of sarcoidosis patients by sex

	**Sex**		**p-value**
	**Male** n=594 (55.5%)	**Female** n=398 (37.2%)	NS
**Sex – Missing data**	N=78 (7.3%)		

Age, years (median [IQR])	52 [42–61]	56 [46–63]	**0.002**
**Smoking status**			
Current/Ex-Smoker (%)	133 (22.4%)	73 (18.3%)	**0.013**
Never (%)	201 (33.8%)	167 (41.9%)	**0.013**
Missing data (%)	260 (43.8%)	158 (39.7%)	NS
**Symptoms**			
Breathlessness (%)	150 (25.2%)	122 (30.6%)	NS
Cough (%)	130 (21.9%)	101 (25.4%)	NS
Fatigue (%)	67 (11.3%)	63 (15.8%)	**0.05**
Erythema nodosum (%)	31 (5.2%)	32 (8.0%)	NS
None (%)	56 (9.4%)	29 (7.3%)	NS
Missing data (%)	266 (44.8%)	160 (40.2%)	NS
**Duration of symptoms prior to chest clinic**			
<6 months (%)	6 (1.0%)	9 (2.2%)	**0.040**
6-12 months (%)	7 (1.2%)	3 (0.7%)	NS
12-24 months (%)	5 (0.8%)	4 (1.0%)	NS
>24 months (%)	7 (1.2%)	5 (1.3%)	NS
Not known (%)	25 (4.2%)	11 (2.8%)	NS
Missing data (%)	545 (91.9%)	366 (91.9%)	NS
**Comorbidities**			
Diabetes (%)	47 (7.9%)	25 (6.3%)	NS
Hypertension (%)	46 (7.7%)	39 (9.8%)	NS
Ischemic heart disease (%)	15 (2.5%)	5 (1.2%)	NS
Arrythmia (%)	6 (1.0%)	0 (0.0%)	**0.042**
Tuberculosis (%)	1 (0.2%)	7 (1.7%)	**0.009**
No comorbidities (%)	169 (28.4%)	116 (29.1%)	NS
Missing data (%)	329 (55.4%)	213 (53.5%)	NS
**Lung function**			
FVC % (median [IQR])	96.1 [91.8-97.0] N= 241 (40.6%)	100.4 [95.1-102.1] N= 151 (37.9%)	NS
DLCO % (median [IQR)	82.7 [78.2-83.0] N= 241 (40.6%)	77.7 [74.3-80.7] N151 (37.9%)	NS
**Blood test results**			
Lymphopenia (%)	118 (19.9%)	56 (14.1%)	**0.002**
Abnormal liver function (%)	52 (8.7%)	28 (7.0%)	NS
Raised ACE (%)	51 (8.6%)	24 (6.0%)	**0.05**
Raised ESR (%)	11 (1.8%)	21 (5.3%)	**0.003**
No abnormalities (%)	335 (56.4%)	218 (54.8%)	NS
Not recorded (%)	43 (7.2%)	47 (11.8%)	NS
**Radiological findings**			
Nodules (%)	166 (27.9%)	129 (32.4%)	NS
Ground glass density (%)	25 (4.2%)	26 (6.5%)	NS
Traction bronchiectasis (%)	24 (4.0%)	14 (3.5%)	NS
Normal (%)	18 (3.0%)	14 (3.5%)	NS
Consolidation (%)	7 (1.2%)	9 (2.3%)	NS
Reticulation (%)	3 (0.5%)	3 (0.7%)	NS
Honeycombing (%)	4 (0.7%)	1 (0.2%)	NS
Cysts (%)	1 (0.2%)	3 (0.7%)	NS
No abnormalities (%)	445 (74.9%)	239 (60.0%)	NS

**Treatment**			
1st line - glucorticosteroids (%)	141 (23.7%)	98 (24.6%)	NS
2nd line - immunosuppressants (%)	40 (6.7%)	20 (5.0%)	NS
Methotrexate (%)	29 (4.9%)	9 (2.3%)	**0.017**
No treatment (%)	204 (34.3%)	141 (35.4%)	NS
Missing data (%)	241 (40.6%)	151 (37.9%)	NS
**Pulmonary rehabilitation**			
Referred (%)	50 (8.4%)	26 (6.5%)	NS
Not assessed (%)	67 (11.3%)	53 (13.3%)	NS
Missing data (%)	477 (80.3%)	319 (80.1%)	NS
**MDT discussion**			
Yes (%)	46 (7.7%)	24 (6.0%)	NS
No (%)	5 (0.8%)	5 (1.2%)	NS
Not documented (%)	543 (91.3)	369 (92.7%)	NS
**Clinical trials**			
Yes (%)	8 (1.3%)	5 (1.2%)	NS
No (%)	259 (43.6%)	182 (45.7%)	NS
Missing data (%)	327 (55.0%)	211 (53.0%)	NS
**Index of Multiple Deprivation Quintiles (IMQ)**			
IMQ1 most deprived (%)	102 (17.2%)	83 (20.8%)	NS
IMQ5 least deprived (%)	72 (12.1%)	60 (15.1%)	NS

Lung-function *n* counts represent participants with data available; those with missing values are excluded from denominators for those rows. MDT – multidisciplinary team, NS – Not Significant

Supplementary Table S3. Descriptive characteristics by detailed ethnicity (transparency table)

Domain	Measure	White	Black African	Black Caribbean	South Asian (Ind/Pak/Bangl.)	East Asian/Chinese	Mixed	Non-White (overall)
Baseline	Number of records	594 (66.89%)	62 (6.98%)	69 (7.77%)	69	1 (0.11%)	20 (2.25%)	142 (15.99%
	Male, n (%)	364 (61.3%)	26 (41.9%)	38 (55.1%)	N/A	1 (100%)	9 (45.0%)	71 (50.0%)
	Female, n (%)	230 (38.7%)	36 (58.1%)	31 (44.9%)	N/A	0 (0%)	11 (55.0%)	71 (50.0%)
	Age at presentation, mean (SD), y	53.18 (13.44)	48.48 (13.86)	49.57 (12.20)	48.40 (12.32)	56.00	N/A	49.20 (12.90
Smoking	Current, n (%)	26 (61.9%)	9 (21.4%)	2 (4.8%)	N/A	N/A	16 (4.8%)	12 (28.6%)
	Ex-smoker, n (%)	117 (74.5%)	12 (8.0%)	8 (5.1%)	N/A	N/A	2 (1.3%)	21 (13.4%)
	Never, n (%)	213 (71.5%)	15 (5.4%)	31 (10.4%)	N/A	N/A	8 (2.7%)	51 (17.1%)
	Not known, n (%)	26 (43.3%)	9 (15.3%)	4 (6.7%)	N/A	N/A	4 (6.7%)	15 (25.0%)
Comorbidities	None	196 (70.3%)	17 (6.4%)	21 (7.5%)	N/A	N/A	6 (2.2%)	41 (14.7%)
	Diabetes	42 (61.8%)	9 (14.3%)	8 (11.8%)	N/A	N/A	3 (4.4%)	19 (27.9%)
	Hypertension	56 (68.3%)	7 (8.9%)	7 (8.5%)	N/A	N/A	1 (1.2%)	15 (18.3%)
	Malignancy	4 (57.1%)	1 (14.3%)	0 (0.0%)	N/A	N/A	0 (0.0%)	1 (14.3%)
	Ischaemic heart disease	14 (70.0%)	0 (0.0%)	5 (25.0%)	N/A	N/A	1 (5.0%)	6 (30.0%)
	Arrhythmia	4 (66.7%)	0 (0.0%)	0 (0.0%)	N/A	N/A	0 (0.0%)	0 (0.0%)
	GERD	11 (91.7%)	1 (8.3%)	0 (0.0%)	N/A	N/A	0 (0.0%)	1 (8.3%)
	Tuberculosis	2 (25.0%)	3 (37.5%)	3 (37.5%)	N/A	N/A	0 (0.0%)	6 (75.0%)
	Depression	3 (50.0%)	1 (16.7%)	1 (16.7%)	N/A	N/A	0 (0.0%)	2 (33.3%)

Lung function	FVC, L (mean, SD)	3.775 (1.140)	3.420 (1.152)	3.001 (1.058)	N/A	N/A	3.723 (1.098)	3.367 (1.158)
	FVC % predicted (mean, SD)	97.00 (20.52)	98.33 (15.06)	85.81 (23.61)	N/A	N/A	102.24 (12.04)	96.32 (18.00)
	DLCO (mean, SD)	7.349 (2.411)	6.596 (2.301)	5.803 (1.914)	N/A	N/A	7.446 (2.234)	6.507 (2.179)
	DLCO % predicted (mean, SD)	80.03 (19.39)	74.12 (16.57)	68.97 (19.31)	N/A	N/A	84.86 (17.13)	74.64 (17.84)
Duration	< 6 months	9 (60.0%)	2 (13.3%)	0 (0.0%)	N/A	N/A	2 (13.3%)	2 (13.3%)
	6–12 months	8 (88.9%)	1 (11.1%)	0 (0.0%)	N/A	N/A	0 (0.0%)	1 (11.1%)
	12–24 months	6 (66.7%)	1 (11.1%)	0 (0.0%)	N/A	N/A	0 (0.0%)	1 (11.1%)
	> 24 months	9 (75.0%)	1 (8.3%)	1 (8.3%)	N/A	N/A	0 (0.0%)	1 (8.3%)
Symptoms	Subcutaneous nodules	13 (65.0%)	3 (15.0%)	1 (5.0%)	N/A	N/A	0 (0.0%)	4 (20.0%)
	Musculoskeletal pain	58 (64.4%)	9 (10.2%)	5 (5.6%)	N/A	N/A	1 (1.1%)	15 (16.7%)
	Other	67 (62.0%)	16 (15.5%)	8 (7.4%)	N/A	N/A	6 (5.6%)	26 (24.1%)
	Fatigue	84 (66.7%)	8 (17.8%)	12 (9.5%)	N/A	N/A	5 (4.0%)	22 (17.5%)
	Eye symptoms	44 (62.9%)	8 (17.8%)	5 (7.1%)	N/A	N/A	3 (4.3%)	15 (21.4%)
	Erythema nodosum	39 (61.9%)	10 (16.1%)	5 (7.9%)	N/A	N/A	1 (1.6%)	16 (25.4%)
	Not known	4 (66.7%)	0 (0.0%)	1 (16.7%)	N/A	N/A	0 (0.0%)	1 (16.7%)
	None	56 (68.3%)	3 (3.9%)	9 (11.0%)	N/A	N/A	3 (3.7%)	14 (17.1%)
	Neurological symptoms	17 (73.9%)	2 (9.1%)	1 (4.3%)	N/A	N/A	1 (4.3%)	3 (13.0%)
	Cardiac symptoms	15 (62.5%)	3 (13.0%)	2 (8.3%)	N/A	N/A	0 (0.0%)	5 (20.8%)
	Fever	11 (55.0%)	3 (17.6%)	4 (20.0%)	N/A	N/A	2 (10.0%)	7 (35.0%)
	Breathlessness	197 (73.8%)	15 (5.9%)	20 (7.5%)	N/A	N/A	8 (3.0%)	40 (15.0%)
	Cough	163 (70.9%)	22 (10.1%)	20 (8.7%)	N/A	N/A	5 (2.2%)	44 (19.1%)
Laboratory	Raised IgG	4 (44.4%)	0 (0.0%)	1 (11.1%)	N/A	N/A	1 (11.1%)	1 (11.1%)
	Other (non-specified)	46 (63.0%)	9 (12.7%)	6 (8.2%)	N/A	N/A	2 (2.7%)	17 (23.3%)
	Raised ESR	24 (75.0%)	4 (13.3%)	3 (9.4%)	N/A	N/A	1 (3.1%)	7 (21.9%)
	Raised CRP	32 (72.7%)	5 (11.9%)	3 (6.8%)	N/A	N/A	0 (0.0%)	8 (18.2%)
	Raised Ca^{2+}	14 (63.6%)	4 (19.0%)	1 (4.5%)	N/A	N/A	0 (0.0%)	5 (22.7%)
	Abnormal liver function	55 (68.8%)	12 (15.4%)	3 (3.8%)	N/A	N/A	2 (2.5%)	16 (20.0%)
	Raised ACE	52 (73.2%)	6 (8.5%)	0 (0.0%)	N/A	N/A	2 (2.8%)	7 (9.9%)
	Raised eosinophils	5 (83.3%)	1 (16.7%)	0 (0.0%)	N/A	N/A	0 (0.0%)	1 (16.7%)
	Abnormal renal function	10 (83.3%)	0 (0.0%)	1 (8.3%)	N/A	N/A	0 (0.0%)	1 (8.3%)
	Raised auto-antibodies	4 (50.0%)	1 (12.5%)	0 (0.0%)	N/A	N/A	0 (0.0%)	1 (12.5%)
	Not recorded	48 (54.5%)	7 (8.4%)	7 (8.0%)	N/A	N/A	7 (8.0%)	20 (22.7%)
	Lymphopenia	141 (81.5%)	7 (4.2%)	10 (5.8%)	N/A	N/A	1 (0.6%)	17 (9.8%)
	Low platelets	3 (75.0%)	0 (0.0%)	0 (0.0%)	N/A	N/A	0 (0.0%)	0 (0.0%)
Radiology (HRCT)	Traction bronchiectasis	26 (70.3%)	4 (11.1%)	1 (2.7%)	N/A	N/A	1 (2.7%)	6 (16.2%)
	Reticulation	3 (50.0%)	1 (16.7%)	0 (0.0%)	N/A	N/A	0 (0.0%)	1 (16.7%)
	Cysts	2 (50.0%)	1 (25.0%)	0 (0.0%)	N/A	N/A	0 (0.0%)	1 (25.0%)
	Honeycombing	5 (100.0%)	0 (0.0%)	0 (0.0%)	N/A	N/A	0 (0.0%)	0 (0.0%)
	Normal	23 (74.2%)	1 (3.4%)	4 (12.9%)	N/A	N/A	0 (0.0%)	5 (16.1%)
	Nodules	207 (71.6%)	20 (7.2%)	24 (8.3%)	N/A	N/A	8 (2.8%)	49 (17.0%)
	Ground-glass opacities	34 (69.4%)	5 (10.9%)	6 (12.2%)	N/A	N/A	1 (2.0%)	11 (22.4%)
	Consolidation	14 (87.5%)	0 (0.0%)	2 (12.5%)	N/A	N/A	0 (0.0%)	2 (12.5%)
Treatment	None	233 (69.8%)	22 (6.9%)	30 (9.0%)	N/A	N/A	9 (2.7%)	55 (16.5%)
	Prednisolone – high dose	140 (67.6%)	18 (9.1%)	16 (7.7%)	N/A	N/A	5 (2.4%)	39 (18.8%)
	Prednisolone – low dose	5 (55.6%)	1 (12.5%)	1 (11.1%)	N/A	N/A	0 (0.0%)	2 (22.2%)
	IV methylprednisolone	3 (42.9%)	0 (0.0%)	2 (28.6%)	N/A	N/A	0 (0.0%)	2 (28.6%)
	Azathioprine	3 (50.0%)	0 (0.0%)	2 (33.3%)	N/A	N/A	0 (0.0%)	2 (33.3%)
	Methotrexate	25 (67.6%)	1 (2.8%)	2 (5.4%)	N/A	N/A	1 (2.7%)	4 (10.8%)
	Hydroxychloroquine	6 (66.7%)	1 (12.5%)	1 (11.1%)	N/A	N/A	1 (11.1%)	3 (33.3%)
	Mycophenolate	2 (28.6%)	3 (42.9%)	0 (0.0%)	N/A	N/A	0 (0.0%)	3 (42.9%)
Oxygen therapy	Not assessed	13 (52.0%)	1 (4.0%)	0 (0.0%)	N/A	N/A	2 (8.0%)	2 (8.0%)
	Not required/appropriate	22 (78.6%)	4 (14.3%)	0 (0.0%)	N/A	N/A	0 (0.0%)	4 (14.3%)
	Referred/received	2 (50.0%)	0 (0.0%)	0 (0.0%)	N/A	N/A	0 (0.0%)	0 (0.0%)
	Not assessed	67 (56.3%)	9 (7.7%)	9 (7.6%)	N/A	N/A	5 (4.2%)	21 (17.6%)

Pulmonary rehabilitation	Referred/Not suitable	46 (80.7%)	4 (7.3%)	3 (5.3%)	N/A	N/A	0 (0.0%)	7 (12.3%)
	Referred/received	8 (72.7%)	0 (0.0%)	2 (18.2%)	N/A	N/A	0 (0.0%)	2 (18.2%)
	Patient declined	3 (100.0%)	0 (0.0%)	0 (0.0%)	N/A	N/A	0 (0.0%)	0 (0.0%)
Clinical trials	Not known	18 (47.4%)	1 (2.8%)	5 (13.2%)	N/A	N/A	1 (2.6%)	7 (18.4%)
	No	309 (72.3%)	32 (7.9%)	37 (8.6%)	N/A	N/A	11 (2.6%)	75 (17.5%)
	Yes recruited	6 (46.2%)	4 (30.8%)	1 (7.7%)	N/A	N/A	0 (0.0%)	5 (38.5%)
MDT	Yes	39 (57.4%)	8 (11.9%)	2 (2.9%)	N/A	N/A	4 (5.9%)	12 (17.6%)
	No	7 (70.0%)	0 (0.0%)	0 (0.0%)	N/A	N/A	0 (0.0%)	0 (0.0%)
	Not known	351 (68.2%)	38 (7.9%)	47 (9.3%)	N/A	N/A	12 (2.4%)	92 (18.2%)
IMD quintiles	Q1 (most deprived)	112 (61.2%)	28 (15.8%)	19 (10.4%)	N/A	N/A	4 (2.2%)	50 (27.3%)
IMD quintiles	Q5 (least deprived)	85 (71.4%)	3 (2.6%)	6 (5.0%)	N/A	N/A	5 (4.2%)	9 (7.6%)

Lung-function *n* counts represent participants with data available; those with missing values are excluded from denominators for those rows

Notes:

1) Denominators vary by row and reflect records with data available; subgroup totals may not exactly equal aggregate counts due to varying completeness.

2) N/A indicates values not available in the provided export.

Supplementary table 4. Comparison of characteristics of sarcoidosis patients by ethnicity – White vs Non-White

	**Ethnicity**		**p-value**
	**White** N=594 (55.5%)	**Non-White** N=152 (14.2%)	**<0.001**
**Ethnicity – Missing data**	N=153		
**Ethnicity – Not stated**	N=172		
**Age, years (median, [IQR])**	54 [44–62]	52 [42–61]	**<0.001**
**Smoking status**			
Current/Ex-Smoker (%)	143 (24.1%)	56 (10.2%)	**<0.001**
Never (%)	213 (35.8%)	85 (15.7%)	**<0.001**
Not Known (%)	26 (11.3%)	34 (12.0%)	**<0.001**
Missing data (%)	55 (9.2%)	37 (24.3%)	**<0.001**
**Symptoms**			
Breathlessness (%)	197 (33.2%)	70 (46.0%)	**0.003**
Cough (%)	163 (27.4%)	67 (44.1%)	**<0.001**
Fatigue (%)	84 (14.1%)	42 (27.6%)	**<0.001**
Erythema nodosum (%)	39 (6.6%)	24 (15.8%)	**<0.001**

Fever (%)	11 (1.8%)	9 (5.9%)	**0.004**
None (%)	56 (9.4%)	26 (17.1%)	**0.006**
Missing data (%)	114 (19.2%)	56 (36.8%)	NS
**Duration of symptoms prior to chest clinic**			
<6 months (%)	9 (1.5%)	6 (3.9%)	**0.05**
6-12 months (%)	8 (1.0%)	1 (0.6%)	NS
12-24 months (%)	6 (1.0%)	3 (2.0%)	NS
>24 months (%)	9 (1.5%)	3 (2.0%)	NS
Missing data (%)	577 (97.1%)	139 (91.4%)	**<0.001**
**Comorbidities**			
Hypertension (%)	56 (9.4%)	26 (18.3%)	**0.004**
Diabetes (%)	42 (7.1%)	26 (18.3%)	**<0.001**
Ischemic heart disease (%)	14 (2.3%)	6 (4.2%)	NS
Arrythmia (%)	4 (0.7%)	2 (1.4%)	NS
Tuberculosis (%)	2 (0.3%)	6 (4.2%)	**<0.001**
None (%)	196 (33.0%)	83 (58.4%)	**<0.001**
Missing data (%)	264 (44.4%)	31 (21.8)	**<0.001**
**Blood test results**			
Lymphopenia (%)	141 (23.7%)	32 (21.0%)	NS
Abnormal liver function (%)	55 (9.2%)	25 (16.4%)	**0.009**
Raised ACE (%)	52 (8.7%)	19 (12.5%)	NS
Raised ESR (%)	24 (4.0%)	8 (5.3%)	NS
No abnormalities (%)	389 (65.5%)	68 (17.1%)	**0.004**
**Radiological findings**			
Nodules (%)	213 (35.8%)	82 (53.9%)	**0.013**
Ground glass density (%)	36 (6.1%)	15 (10.0%)	NS
Traction bronchiectasis (%)	26 (4.4%)	12 (7.9%)	NS
Normal (%)	23 (3.9%)	9 (5.9%)	NS
Consolidation (%)	14 (2.3%)	2 (1.3%)	NS
Reticulation (%)	3 (0.5%)	3 (2.0%)	NS
Honeycombing (%)	5 (0.8%)	0 (0.0%)	NS
Cysts (%)	2 (0.3%)	2 (1.3%)	NS
No abnormalities (%)	262 (44.1%)	27 (17.8%)	**<0.001**
**Lung function**			
FVC % (median, [IQR))	98.4 [94.6-99.4] N= 247 (41.6%)	98.4 [90.5-98.4] N= 110 (72.4%)	NS
DLCO % (median, [IQR])	80.0 [77.7-82.3] N= 247 (41.6%)	77.7 [74.1-81.1] N= 110 (72.4%)	NS

**Treatment**			
1st line - glucorticosteroids (%)	157 (26.4%)	77 (23.8%)	NS
2nd line - immunosuppressants (%)	37 (6.2%)	22 (6.8%)	NS
Methotrexate (%)	25 (4.2%)	12 (6.5%)	NS
Mycophenolate mofetil (%)	2 (0.3%)	5 (3.7%)	**0.036**
No treatment (%)	233 (39.2%)	45 (29.6%)	NS
Missing data (%)	101 (17.0%)	8 (5.3%)	NS
**Pulmonary rehabilitation**			
Referred (%)	8 (1.3%)	3 (0.9%)	NS
Not assessed (%)	67 (11.3%)	52 (16.0%)	**0.042**
Not known (%)	324 (54.5)	80 (52.6%)	NS
Missing data (%)	195(32.8%)	17 (11.2%)	**0.003**
**MDT discussion**			
Yes (%)	39 (6.6%)	29 (19.1%)	**0.032**
No (%)	7 (1.2%)	3 (2.0%)	NS
Not documented (%)	548 (92.2%)	120 (78.9%)	NS
**Clinical trials**			
Yes (%)	6 (1.0%)	7 (2.2%)	NS
No (%)	310 (52.2%)	119 (36.7%)	**<0.001**
Not known (%)	18 (3.0%)	20 (6.2%)	**0.019**
Missing data (%)	260 (43.8%)	6 (3.9%)	**0.001**
**Index of Multiple Deprivation Quintiles (IMQ)**			
IMQ1 most deprived (%)	112 (18.8%)	71 (21.9%)	**0.022**
IMQ5 least deprived (%)	85 (14.3%)	34 (10.5%)	0.081

Lung-function *n* counts represent participants with data available; those with missing values are excluded from denominators for those rows
