# Supplementary material for: Exploring gender and ethnic disparities in sarcoidosis: insights from the British Thoracic Society UK Interstitial Lung Disease Registry
Source: BMJ Open Respir Res. 2025 Nov 18;12(1):e003449. doi: 10.1136/bmjresp-2025-003449 (PMC12636952; doi:10.1136/bmjresp-2025-003449)
Supplement: online supplemental file 2 [file bmjresp-12-1-s002.pdf]

Supplementary Table S3. Descriptive characteristics by detailed ethnicity (transparency table)

Domain	Measure	White	Black African	Black Caribbean	South Asian (Ind/Pak/Bangl.)	East Asian/Chinese	Mixed	Non-White (overall)
Baseline	Number of records	594 (66.89%)	62 (6.98%)	69 (7.77%)	69	1 (0.11%)	20 (2.25%)	142 (15.99%)
	Male, n (%)	364 (61.3%)	26 (41.9%)	38 (55.1%)	N/A	1 (100%)	9 (45.0%)	71 (50.0%)
	Female, n (%)	230 (38.7%)	36 (58.1%)	31 (44.9%)	N/A	0 (0%)	11 (55.0%)	71 (50.0%)
	Age at presentation, mean (SD), y	53.18 (13.44)	48.48 (13.86)	49.57 (12.20)	48.40 (12.32)	56.00	N/A	49.20 (12.90)
Smoking	Current, n (%)	26 (61.9%)	9 (21.4%)	2 (4.8%)	N/A	N/A	16 (4.8%)	12 (28.6%)
	Ex-smoker, n (%)	117 (74.5%)	12 (8.0%)	8 (5.1%)	N/A	N/A	2 (1.3%)	21 (13.4%)
	Never, n (%)	213 (71.5%)	15 (5.4%)	31 (10.4%)	N/A	N/A	8 (2.7%)	51 (17.1%)
	Not known, n (%)	26 (43.3%)	9 (15.3%)	4 (6.7%)	N/A	N/A	4 (6.7%)	15 (25.0%)
Comorbidities	None	196 (70.3%)	17 (6.4%)	21 (7.5%)	N/A	N/A	6 (2.2%)	41 (14.7%)
	Diabetes	42 (61.8%)	9 (14.3%)	8 (11.8%)	N/A	N/A	3 (4.4%)	19 (27.9%)
	Hypertension	56 (68.3%)	7 (8.9%)	7 (8.5%)	N/A	N/A	1 (1.2%)	15 (18.3%)
	Malignancy	4 (57.1%)	1 (14.3%)	0 (0.0%)	N/A	N/A	0 (0.0%)	1 (14.3%)
	Ischaemic heart disease	14 (70.0%)	0 (0.0%)	5 (25.0%)	N/A	N/A	1 (5.0%)	6 (30.0%)
	Arrhythmia	4 (66.7%)	0 (0.0%)	0 (0.0%)	N/A	N/A	0 (0.0%)	0 (0.0%)
	GERD	11 (91.7%)	1 (8.3%)	0 (0.0%)	N/A	N/A	0 (0.0%)	1 (8.3%)
	Tuberculosis	2 (25.0%)	3 (37.5%)	3 (37.5%)	N/A	N/A	0 (0.0%)	6 (75.0%)

	Depression	3 (50.0%)	1 (16.7%)	1 (16.7%)	N/A	N/A	0 (0.0%)	2 (33.3%)
Lung function	FVC, L (mean, SD)	3.775 (1.140)	3.420 (1.152)	3.001 (1.058)	N/A	N/A	3.723 (1.098)	3.367 (1.158)
	FVC % predicted (mean, SD)	97.00 (20.52)	98.33 (15.06)	85.81 (23.61)	N/A	N/A	102.24 (12.04)	96.32 (18.00)
	DLCO (mean, SD)	7.349 (2.411)	6.596 (2.301)	5.803 (1.914)	N/A	N/A	7.446 (2.234)	6.507 (2.179)
	DLCO % predicted (mean, SD)	80.03 (19.39)	74.12 (16.57)	68.97 (19.31)	N/A	N/A	84.86 (17.13)	74.64 (17.84)
Duration	< 6 months	9 (60.0%)	2 (13.3%)	0 (0.0%)	N/A	N/A	2 (13.3%)	2 (13.3%)
	6–12 months	8 (88.9%)	1 (11.1%)	0 (0.0%)	N/A	N/A	0 (0.0%)	1 (11.1%)
	12–24 months	6 (66.7%)	1 (11.1%)	0 (0.0%)	N/A	N/A	0 (0.0%)	1 (11.1%)
	> 24 months	9 (75.0%)	1 (8.3%)	1 (8.3%)	N/A	N/A	0 (0.0%)	1 (8.3%)
Symptoms	Subcutaneous nodules	13 (65.0%)	3 (15.0%)	1 (5.0%)	N/A	N/A	0 (0.0%)	4 (20.0%)
	Musculoskeletal pain	58 (64.4%)	9 (10.2%)	5 (5.6%)	N/A	N/A	1 (1.1%)	15 (16.7%)
	Other	67 (62.0%)	16 (15.5%)	8 (7.4%)	N/A	N/A	6 (5.6%)	26 (24.1%)
	Fatigue	84 (66.7%)	8 (17.8%)	12 (9.5%)	N/A	N/A	5 (4.0%)	22 (17.5%)
	Eye symptoms	44 (62.9%)	8 (17.8%)	5 (7.1%)	N/A	N/A	3 (4.3%)	15 (21.4%)
	Erythema nodosum	39 (61.9%)	10 (16.1%)	5 (7.9%)	N/A	N/A	1 (1.6%)	16 (25.4%)
	Not known	4 (66.7%)	0 (0.0%)	1 (16.7%)	N/A	N/A	0 (0.0%)	1 (16.7%)
	None	56 (68.3%)	3 (3.9%)	9 (11.0%)	N/A	N/A	3 (3.7%)	14 (17.1%)
	Neurological symptoms	17 (73.9%)	2 (9.1%)	1 (4.3%)	N/A	N/A	1 (4.3%)	3 (13.0%)

	Cardiac symptoms	15 (62.5%)	3 (13.0%)	2 (8.3%)	N/A	N/A	0 (0.0%)	5 (20.8%)
	Fever	11 (55.0%)	3 (17.6%)	4 (20.0%)	N/A	N/A	2 (10.0%)	7 (35.0%)
	Breathlessness	197 (73.8%)	15 (5.9%)	20 (7.5%)	N/A	N/A	8 (3.0%)	40 (15.0%)
	Cough	163 (70.9%)	22 (10.1%)	20 (8.7%)	N/A	N/A	5 (2.2%)	44 (19.1%)
Laboratory	Raised IgG	4 (44.4%)	0 (0.0%)	1 (11.1%)	N/A	N/A	1 (11.1%)	1 (11.1%)
	Other (non-specified)	46 (63.0%)	9 (12.7%)	6 (8.2%)	N/A	N/A	2 (2.7%)	17 (23.3%)
	Raised ESR	24 (75.0%)	4 (13.3%)	3 (9.4%)	N/A	N/A	1 (3.1%)	7 (21.9%)
	Raised CRP	32 (72.7%)	5 (11.9%)	3 (6.8%)	N/A	N/A	0 (0.0%)	8 (18.2%)
	Raised Ca^{2+}	14 (63.6%)	4 (19.0%)	1 (4.5%)	N/A	N/A	0 (0.0%)	5 (22.7%)
	Abnormal liver function	55 (68.8%)	12 (15.4%)	3 (3.8%)	N/A	N/A	2 (2.5%)	16 (20.0%)
	Raised ACE	52 (73.2%)	6 (8.5%)	0 (0.0%)	N/A	N/A	2 (2.8%)	7 (9.9%)
	Raised eosinophils	5 (83.3%)	1 (16.7%)	0 (0.0%)	N/A	N/A	0 (0.0%)	1 (16.7%)
	Abnormal renal function	10 (83.3%)	0 (0.0%)	1 (8.3%)	N/A	N/A	0 (0.0%)	1 (8.3%)
	Raised auto-antibodies	4 (50.0%)	1 (12.5%)	0 (0.0%)	N/A	N/A	0 (0.0%)	1 (12.5%)
	Not recorded	48 (54.5%)	7 (8.4%)	7 (8.0%)	N/A	N/A	7 (8.0%)	20 (22.7%)
	Lymphopenia	141 (81.5%)	7 (4.2%)	10 (5.8%)	N/A	N/A	1 (0.6%)	17 (9.8%)
	Low platelets	3 (75.0%)	0 (0.0%)	0 (0.0%)	N/A	N/A	0 (0.0%)	0 (0.0%)
Radiology (HRCT)	Traction bronchiectasis	26 (70.3%)	4 (11.1%)	1 (2.7%)	N/A	N/A	1 (2.7%)	6 (16.2%)

	Reticulation	3 (50.0%)	1 (16.7%)	0 (0.0%)	N/A	N/A	0 (0.0%)	1 (16.7%)
	Cysts	2 (50.0%)	1 (25.0%)	0 (0.0%)	N/A	N/A	0 (0.0%)	1 (25.0%)
	Honeycombing	5 (100.0%)	0 (0.0%)	0 (0.0%)	N/A	N/A	0 (0.0%)	0 (0.0%)
	Normal	23 (74.2%)	1 (3.4%)	4 (12.9%)	N/A	N/A	0 (0.0%)	5 (16.1%)
	Nodules	207 (71.6%)	20 (7.2%)	24 (8.3%)	N/A	N/A	8 (2.8%)	49 (17.0%)
	Ground-glass opacities	34 (69.4%)	5 (10.9%)	6 (12.2%)	N/A	N/A	1 (2.0%)	11 (22.4%)
	Consolidation	14 (87.5%)	0 (0.0%)	2 (12.5%)	N/A	N/A	0 (0.0%)	2 (12.5%)
Treatment	None	233 (69.8%)	22 (6.9%)	30 (9.0%)	N/A	N/A	9 (2.7%)	55 (16.5%)
	Prednisolone – high dose	140 (67.6%)	18 (9.1%)	16 (7.7%)	N/A	N/A	5 (2.4%)	39 (18.8%)
	Prednisolone – low dose	5 (55.6%)	1 (12.5%)	1 (11.1%)	N/A	N/A	0 (0.0%)	2 (22.2%)
	IV methylprednisolone	3 (42.9%)	0 (0.0%)	2 (28.6%)	N/A	N/A	0 (0.0%)	2 (28.6%)
	Azathioprine	3 (50.0%)	0 (0.0%)	2 (33.3%)	N/A	N/A	0 (0.0%)	2 (33.3%)
	Methotrexate	25 (67.6%)	1 (2.8%)	2 (5.4%)	N/A	N/A	1 (2.7%)	4 (10.8%)
	Hydroxychloroquine	6 (66.7%)	1 (12.5%)	1 (11.1%)	N/A	N/A	1 (11.1%)	3 (33.3%)
	Mycophenolate	2 (28.6%)	3 (42.9%)	0 (0.0%)	N/A	N/A	0 (0.0%)	3 (42.9%)
Oxygen therapy	Not assessed	13 (52.0%)	1 (4.0%)	0 (0.0%)	N/A	N/A	2 (8.0%)	2 (8.0%)
	Not required/appropriate	22 (78.6%)	4 (14.3%)	0 (0.0%)	N/A	N/A	0 (0.0%)	4 (14.3%)
	Referred/received	2 (50.0%)	0 (0.0%)	0 (0.0%)	N/A	N/A	0 (0.0%)	0 (0.0%)

Pulmonary rehabilitation	Not assessed	67 (56.3%)	9 (7.7%)	9 (7.6%)	N/A	N/A	5 (4.2%)	21 (17.6%)
	Referred/Not suitable	46 (80.7%)	4 (7.3%)	3 (5.3%)	N/A	N/A	0 (0.0%)	7 (12.3%)
	Referred/received	8 (72.7%)	0 (0.0%)	2 (18.2%)	N/A	N/A	0 (0.0%)	2 (18.2%)
	Patient declined	3 (100.0%)	0 (0.0%)	0 (0.0%)	N/A	N/A	0 (0.0%)	0 (0.0%)
Clinical trials	Not known	18 (47.4%)	1 (2.8%)	5 (13.2%)	N/A	N/A	1 (2.6%)	7 (18.4%)
	No	309 (72.3%)	32 (7.9%)	37 (8.6%)	N/A	N/A	11 (2.6%)	75 (17.5%)
	Yes recruited	6 (46.2%)	4 (30.8%)	1 (7.7%)	N/A	N/A	0 (0.0%)	5 (38.5%)
MDT	Yes	39 (57.4%)	8 (11.9%)	2 (2.9%)	N/A	N/A	4 (5.9%)	12 (17.6%)
	No	7 (70.0%)	0 (0.0%)	0 (0.0%)	N/A	N/A	0 (0.0%)	0 (0.0%)
	Not known	351 (68.2%)	38 (7.9%)	47 (9.3%)	N/A	N/A	12 (2.4%)	92 (18.2%)
IMD quintiles	Q1 (most deprived)	112 (61.2%)	28 (15.8%)	19 (10.4%)	N/A	N/A	4 (2.2%)	50 (27.3%)
IMD quintiles	Q5 (least deprived)	85 (71.4%)	3 (2.6%)	6 (5.0%)	N/A	N/A	5 (4.2%)	9 (7.6%)
